# Blockchain Factors in the Design of Smart-Media for E-Healthcare Management

**DOI:** 10.3390/s24216835

**Published:** 2024-10-24

**Authors:** Dhaneshwar Shah, Sunanda Rani, Khadija Shoukat, Habiba Kalsoom, Muhammad Usman Shoukat, Hamad Almujibah, Shengxiao Liao

**Affiliations:** 1 School of Art & Design, Wuhan University of Technology, Wuhan 430070, China; sunanda2017@whut.edu.cn; 2 School of Management, Wuhan University of Technology, Wuhan 430062, China; ryk2022@whut.edu.cn; 3 Department of Zoology, The Government Sadiq College Women University, Bahawalpur 63100, Pakistan; habiba31205@gmail.com; 4 School of Automotive Engineering, Wuhan University of Technology, Wuhan 430070, China; usmanryk12@gmail.com; 5 Department of Civil Engineering, College of Engineering, Taif University, Taif 21974, Saudi Arabia; hmujibah@tu.edu.sa; 6 The School of International Education, Wuhan University of Technology, Wuhan 430062, China; liaoshengxiao@whut.edu.cn

**Keywords:** blockchain, IoT, e-healthcare, electronic health records, data sharing, data security

## Abstract

According to the current situation of deep aging globally, how to provide low-cost and high-quality medical services has become a problem that the whole society needs to consider. To address these challenges, we propose an e-healthcare management system leveraging the integration of the Internet of Things (IoT) and blockchain technologies. Our system aims to provide comprehensive, reliable, and secure one-stop services for patients. Specifically, we introduce a blockchain-based searchable encryption scheme for decentralized storage and real-time updates of electronic health records (EHRs). This approach ensures secure and efficient data traceability across medical equipment, drug supply chains, patient health monitoring, and medical big data management. By improving information processing capabilities, our system aspires to advance the digital transformation of e-healthcare services.

## 1. Introduction

The deep integration of the internet, big data, the IoT, and other information technologies with conventional medical and health services has created a new type of medical and health service format known as “e-healthcare”. Through the interaction between medical personnel, medical institutions, and medical equipment, we will gradually achieve the intelligent transformation of medical services [1,2,3,4]. However, despite advancements in digital health systems such as hospital information systems (HISs), EMR, and picture archiving communication systems (PACSs), there remain critical challenges: inadequate centralized support for diagnostics, lack of trust between healthcare providers and patients, and difficulties in secure data sharing and management [5,6]. Governments worldwide are prioritizing digital healthcare transformation to address these issues, with a focus on integrating healthcare, medical services, and insurance [7,8,9,10]. Blockchain technology, with its distributed, immutable, and trust-free nature, offers a promising solution to many of these challenges. When combined with the IoT, blockchain can significantly improve data collection, storage, and accessibility, leading to better patient care, enhanced data security, and increased operational efficiency [11,12].

At the same time, data storage is not the property of anyone, is not under the control of users, and is not subject to regulation by any centralized regulatory body or trusted third party [13]. At present, we are entering the era of blockchain 3.0, represented by the alliance chain. Blockchain not only meets the authenticity and integrity requirements of health data but also provides technical means for proactive governance by health supervision departments. The value of “e-healthcare” is the sharing of information resources. However, there is currently a serious problem of information asymmetry between doctors and patients, between medical institutions and management departments, and between medical institutions and insurance departments [14,15]. This characteristic makes it extremely challenging for malicious actors to tamper with or manipulate medical data, ensuring the trustworthiness and authenticity of the information stored on the blockchain.

Despite the progress in e-healthcare, the current systems still struggle with data integrity, privacy management, and trust issues. Blockchain, an integrated application of distributed data storage, peer-to-peer transmission, consensus mechanisms, encryption algorithms, smart contracts, and other technologies, has become a hot topic for public research and industry investment [16,17,18]. Blockchain technology is used to build a comprehensive medical information platform [19,20] and a drug and medical device traceability system [21]. Our proposed e-healthcare management system, built on blockchain and the IoT, aims to tackle these challenges by creating a decentralized, immutable ledger for medical data. This system enhances real-time data collection, monitoring, and analysis through the IoT while ensuring data security and privacy through blockchain. Furthermore, by employing a blockchain-based searchable encryption scheme, our system facilitates the secure storage and updating of EHRs, making it a practical and secure solution for modern healthcare management [22]. The following are the goals of this paper in light of the previously mentioned gaps:

- This study is fully committed to creating cooperation models and a medical complex on the bases of EHRs that integrates clinical services, medical education, medical research, and achievement transformation.
- We are vigorously planning to build a batch of smart medical and smart elderly care industry bases that integrate health, wellness, and elderly care functions [23].
- This study proposes a blockchain- and IoT-based solution to fill gaps in e-healthcare systems. We use empirical examples to show this system’s potential and offer insights into its deployment to improve healthcare.

The subsequent sections of the document are structured in the following way. Section 2 provides a comprehensive overview of previous research conducted on blockchain and the IoT employed in e-healthcare system. In Section 3, the methodology employed for the collection of data is expounded upon. Section 4 of the study presents the requirements and limitations of various statistical analyses, such as interoperability, authenticity, and traceability. Section 5 of the document elaborates on the implementation of an e-healthcare management system and the analysis utilized for data collection. Section 6 of the study presents the results analysis. Ultimately, the outcomes of the study are synthesized, and their potential consequences are outlined in Section 7.

## 2. Related Works

The utilization of blockchain technology has been advocated in recent literature [24] as a means to enhance healthcare administration by enabling the reliable digitization of records into a decentralized database. Through a questionnaire survey, Luo et al. [25] examined the influence of the financial status of the hospital, perceived usefulness, and perceived ease of use on the adoption of health information systems. However, according to McGhin et al. [26], the existing blockchain technology falls short in fulfilling the demands of healthcare applications. Chendeb et al. [27] proposed a blockchain-based architecture for secure and efficient sharing of healthcare data generated by IoT devices. The system utilized blockchain to ensure data integrity, transparency, and access control while leveraging IoT devices for real-time data collection. The study highlighted the benefits of blockchain in maintaining data provenance and enabling secure data sharing across different healthcare providers.

Although blockchain technology holds immense potential for IoT applications, it is currently constrained by various limitations. Ourad et al. [28] have presented a solution and framework based on blockchain technology to ensure secure user authentication for accessing IoT devices. Their proposed architecture has been shown to address the limitations of current authentication methods and provide features such as traceability, integrity, and accountability through the use of immutable logs. The current state of emerging blockchain technology involves the participation of every network node in transaction processing. However, this approach results in inefficiencies and inadequacies that limit its ability to handle real-world applications that require processing rates of tens of thousands of transactions per second [29]. Table 1 lists the top IoT-enabled healthcare system research and their primary contributions and drawbacks.

The advancement of smart contract technology can be attributed to the emergence of blockchain technology. The healthcare data blockchain preserves a complete historical record of all medical data, covering EHR (electronic health record), access, prescription, billing, and IoT data, which will always follow a single user. The usage of blockchain for sharing EHRs can increase patient opt-in rates, as demonstrated by Sharma et al. (2020), who used qualitative data based on soft systems methodology to make their case [40]. Their main priority was the precision health care (PHC) program, which is a database of people’s EHRs for the public good. Chelladurai et al.’s [41] framework implements the topic differently, with an immutable log creation model, patient–provider authorization model for EHR updates, data sharing methodology, and viewership model. The EHR update model manages EHR updates, whereas the immutable log creation approach creates new EHR data. Data sharing and viewership mechanisms govern EHR access. The authors examine how blockchain technology is used in healthcare. The suggested method updates EHRs dynamically. This technique does not handle encryption and keyword search on encrypted EHRs. This authorization model needs entity verification.

Chakraborty et al. [42] explored the application of blockchain technology to enhance the security of medical IoT devices. Shyamala Devi et al. [43] presented a framework that combined the IoT and blockchain to facilitate healthcare monitoring and decision-making. They integrated wearable sensors and smart devices to collect real-time patient data, which were stored on a blockchain network for secure and auditable access. However, the proposed framework did not demonstrate the potential of the IoT and blockchain in enabling personalized healthcare and efficient decision support systems.

Shahnaz et al. (2019) presented a framework aimed at addressing the issue of scalability when utilizing blockchain technology [44]. Li et al. [45] propose integration of collaborative beamforming into the Internet of Things (IoT) and Unmanned Aerial Vehicles (UAVs) to efficiently collect and distribute data from many IoT clusters to far base stations (BSs) in terms of energy and time. Blockchain-based systems have both positive and negative implications for healthcare providers and patients, thus presenting opportunities for further research in this domain [46]. Despite some recent research on the application of blockchain in healthcare management, the precise function of blockchain technology within healthcare systems remains ambiguous [47]. Currently, there are ongoing advancements aimed at enhancing the distinction of e-health commodities. Table 2 provides a comprehensive overview of the current representative resources and approaches available. The present study focuses on the challenges associated with blockchain- and IoT-based e-healthcare management systems. It is noteworthy that this is the first empirical investigation that explores the relationship between blockchain technology and patients’ willingness to disclose their medical records through the mediating effects. Furthermore, there is a dearth of theoretical underpinnings to elucidate the function of extrinsic motivation and security perception in the context of an information system utilized by healthcare providers.

## 3. Methodology

### 3.1. System Architecture Overview

**Figure 1:**
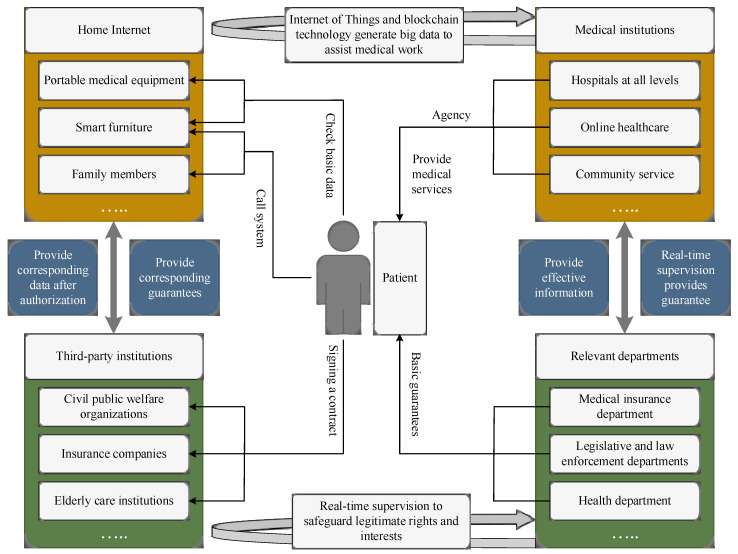
Framework of proposed work: Internet of Things- and blockchain-based e-healthcare system.

With the continuous development of medical information technology, the application of the IoT and blockchain in the medical field is becoming increasingly widespread. We designed an e-healthcare system based on the IoT and blockchain, and the system framework is shown in Figure 1.

The proposed e-healthcare system leverages the combined strengths of the IoT and blockchain to create a secure, decentralized platform for managing patient data. The system architecture is depicted in Figure 1, illustrating the interaction between patients, medical institutions, third-party organizations, and relevant government departments. The system is designed to enhance data interoperability, security, and real-time monitoring through the following components:

IoT infrastructure: the IoT framework consists of three layers:

- Perception layer: This includes RFID tags, portable medical equipment, and smart home devices that collect and transmit patient data. For instance, continuous glucose monitors (CGMs) and wearable ECG monitors fall under this category.
- Network transmission Layer: Data collected from IoT devices are transmitted over secure channels using protocols like Wi-Fi, Bluetooth, and 4G/5G networks. Secure communication protocols such as TLS/SSL are employed to prevent data breaches during transmission.
- Application layer: This layer consists of user interfaces and management platforms that healthcare providers and patients interact with. Blockchain integration at this layer ensures that all interactions are logged and verifiable.

Blockchain integration: The system employs a hybrid blockchain model, incorporating public, alliance, and private chains:

- Public chain: Used for storing non-sensitive data like general health statistics and open-access research findings, ensuring transparency and accessibility.
- Alliance chain: This is used for managing sensitive data that require collaboration between multiple entities, such as medical records shared between hospitals and insurance companies. The alliance chain ensures efficient data exchange while maintaining privacy through controlled access.
- Private chain: Patient-specific data, including financial information and detailed medical histories, are stored on a private chain. Access to this data is tightly controlled through private keys, and modifications are logged via a consensus mechanism to ensure traceability.

Through the IoT and blockchain technology, it covers the data provided by portable medical equipment, smart furniture, etc., and generates big data for the statistics of patient health information, providing corresponding data for the diagnosis and treatment of medical institutions and the work of other institutions. Due to the characteristics of blockchain, such as non-tampering and real-time synchronization, the authenticity and effectiveness of big data on health information are guaranteed. Medical institutions are medical departments at all levels that contract with patients to provide medical solutions for them through joint decision-making based on big data on patient health information. By signing contracts, patients and third-party institutions will put home medical and non-governmental organizations, pension institutions, commercial insurance companies, and other institutions on the chain, making all aspects of information more secure, more decentralized, and more open and transparent. Relevant departments can access the information in each chain of the blockchain, use the security and traceability of the blockchain, supervise all aspects, and protect their reasonable rights and interests. In order to effectively regulate, reduce costs, increase productivity, and safeguard everyone’s rights and interests, the previously mentioned e-healthcare system employs cutting-edge technologies like the IoT, blockchain, and big data [56].

### 3.2. Blockchain- and IoT-Based E-Healthcare Management Systems

**Figure 2:**
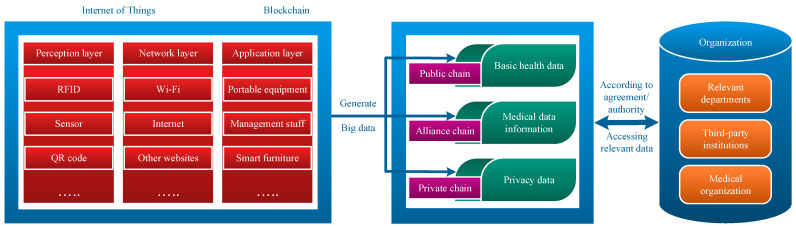
Healthcare management system: IoT and blockchain technology map.

According to different application requirements, the composition architecture of blockchain varies and can be divided into alliance chains, private chains, and public chains according to the degree of decentralization. The biggest characteristics of the public chain are openness and transparency, a high degree of decentralization, and accessibility to any unit. The characteristics of the alliance chain are polycentric, and access units must sign corresponding agreements to obtain access qualifications, resulting in high work efficiency. The characteristic of private chains is weak centralization, which only allows access to internal protocol units and has extremely high security and traceability. The IoT and blockchain technologies of the e-healthcare management system are shown in Figure 2. The proposed e-healthcare system integrates blockchain and IoT technologies to ensure secure, real-time management and sharing of patient health data. The architecture is designed to address various layers of data acquisition, transmission, processing, and application.

The basic health information of patients has reference value in medical research and other aspects, so a public chain architecture is adopted for access by various units. The public chain content of relevant departments, third-party institutions, and medical institutions mainly includes information such as document disclosure, policy interpretation, online consultation, and service guidelines. The EHR owner uploads the index, which is then encrypted and stored in the blockchain. The smart contract owner (the EHR owner) must grant access to the EHR to any organization wishing to conduct a search.

The publishing entity of patient medical data information needs to obtain permission to meet the characteristics of efficiency, so it is linked with an alliance chain for access by medical institutions at all levels and some contracted third-party institutions (such as elderly care institutions). The main content of the alliance chain of third-party institutions (such as community service centers) includes community daily life, six support channels, remote monitoring, nursing management, cost recording, etc. [57]. The alliance chain of medical institutions includes fee settlement, medical examination reports, medical services, medical insurance projects, case archiving, etc. Relevant departments can supervise and manage the alliance chain content of third-party institutions and medical institutions and protect the legitimate rights and interests of all parties in case of problems.

The financial capacity, level of security, family status, and other private information of individual patients require high storage requirements and traceability and need to be linked to a private chain. Some contracted third-party institutions and relevant departments can access privacy information within the scope of the agreement on the private chain.

Blockchain has special distributed ledger technology, and all nodes on the chain can share and maintain data systems to ensure the reliability of data information. At the same time, the consensus mechanism of blockchain technology can allow different departments to participate in verification together. When data need to be modified, consent from other blocks on the chain is required to generate a new blockchain, ensuring the authenticity and traceability of the data.

Applying blockchain technology to e-healthcare management systems can solve problems such as data fraud and insufficient security in existing databases. Through the unique form of the blockchain alliance chain, the e-healthcare management system achieves decentralization and information disclosure. At the same time, institutions with private keys on the alliance chain are also eligible to access relevant data and ensure the privacy of institutions within the system.

## 4. Requirements and Limitations

### 4.1. Interoperability

The sincerity of blockchain determines that its degree of information interconnection is different from the current meaning of “information sharing”. The informatization of hospitals, including patient examinations, payments, and diagnoses, has been digitized, and the globe has invested considerably in this effort. Almost all medical information records are stored in information systems. However, the fragmented medical data environment makes it difficult to meet the immediate needs of patients for medical information, and there is still a big gap between the realization of safe, reliable, and effective sharing of medical information between providers and users. Patients want to be able to view their EHRs and medical records at any time. Medical institutions hope to grasp the past medical history and diagnosis and treatment records of patients (especially those from other places), avoid medical accidents, and provide the best diagnosis and treatment services to patients. Pharmaceutical and medical research institutions hope to establish direct contact with patients involved in research, forming a more efficient and transparent communication mechanism. Unlike traditional databases, the advantage of blockchain is distributed accounting, and all historical records are publicly available, allowing everyone to access and verify relevant records [58,59]. Therefore, it can meet the needs for information sharing mentioned above.

### 4.2. Authenticity

In complex application environments, whether it is online consultation by family doctors or hierarchical diagnosis and treatment by internet hospitals, relying solely on usernames and passwords is far from meeting the needs of personal information protection [60]. Both institutions and individuals need to be strongly authenticated, and a high-security and reliable identity authentication platform should be established to ensure the authenticity and trustworthiness of user identities. When registering electronic medical records of patients and electronic health records of ordinary residents, the user’s identity needs to be verified in real name to ensure the authenticity of the information. When users log in to various health platforms for information queries, strong identity authentication is required. With blockchain as the underlying technology, medical institutions and enterprises can develop various health applications with a variety of user roles and complex responsibility and authority recognition mechanisms. Only by ensuring the credibility of platform user identities can access control effectively be ensured for users, and related businesses can be carried out in an orderly manner.

### 4.3. Traceability

The construction of the “e-healthcare” management system, as a new form of medical service to solve patients’ difficulties in seeking medical treatment and improve medical service experience and efficiency, can improve the uneven distribution of medical resources. In the cross-hospital and cross-regional circulation of medical data, it is necessary to combine reliable electronic signatures with the authenticity of the identities of both doctors and patients, establish an information platform data responsibility recognition mechanism, prevent potential medical disputes in the online medical service process, and ensure the legitimate rights and interests of both doctors and patients.

### 4.4. Limitations

The growth of home-based medical services around the world is currently disorganized, and there is a lack of cooperation between different sectors. There are still substantial obstacles to comprehensive treatment. Here are the most significant issues:

(1) The conversion of medical service locations from hospitals to households increases the safety risks of medical services and limits the development of medical services in terms of medical equipment, medical environment, and other aspects.

(2) There is a very low proportion of experienced and high-level doctors in China compared to home patients. Experienced medical workers mostly work within the medical service system and have no extra time to engage in medical work.

(3) At present, home-based medical services have not been included in the scope of medical insurance reimbursement. Compared with foreign home-based medical services and hospital-centered medical models, home-based medical services in China lack external capital investment, and willingness to pay is relatively low.

(4) The doctor–patient relationship is tense today, and if a medical accident occurs while receiving medical care, the medical service provider is most likely to bear the burden of proof. This concern hinders the development of home medical services.

While the current data focus on encryption efficiency, there is no mention of how the system performs under varying conditions such as different scales of data or varying security requirements. Below is how the results section can be expanded.

## 5. Implementation of E-Healthcare Management System and Analysis

We present the “e-healthcare” model, which represents the proposed scheme, a blockchain- and IoT-based healthcare management system with two-side verifiability. Below, we will go over some of e-healthcare’s key features and procedures.

### 5.1. Promote the Interconnection

Considering the impact of cost input and investment inertia, the blockchain IoT-based medical information system should be built on the existing EHR infrastructure rather than restructured. The solution includes creating a new facility based on blockchain and IoT technology to store clinical data while continuing to use current medical information systems to obtain and store patient data. The existing information system will not only store data in a dedicated database but also transmit a copy to EHRs based on the blockchain. This solution not only retains the advantages of blockchain technology but also utilizes existing achievements in medical information construction. The existing standards and policies provide a framework for replicating data from traditional systems to new blockchain-based systems.

In order to achieve the functions of reading, downloading, and transmitting, data in medical information systems are generally stored and transmitted in the HL7 (health level-7) clinical document structure and are available for people to read in the HTML (hypertext markup language) document format through application style sheets. Two options are proposed below.

**Figure 3:**
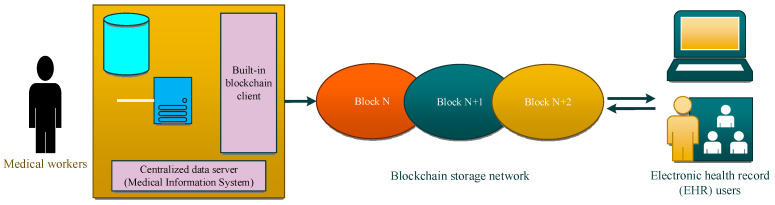
Schematic diagram of medical data exchange process.

Option 1: Install a blockchain client in the existing medical information system to automatically transmit health information to EHRs based on the blockchain, as shown in Figure 3. The patient’s medical information is saved locally, and a copy is generated and uploaded to the blockchain using the HL7 clinical document structure. The blockchain network determines the validity of information through a consensus mechanism, and more than half of the nodes agree to submit it to the general ledger. On the blockchain platform, a distributed ledger keeps track of all patient-generated medical and health data exchange processes in a transparent, secure, and auditable manner. The medical information system can arrange this medical information in chronological order and upload the data to specific categories. This classification not only makes it easier for patients to access and understand EHRs but also facilitates researchers in obtaining information related to them. This solution requires the active participation of the medical information system management platform, as well as supporting supervision and incentive measures to ensure the implementation of the solution.

Option 2: Patients actively participate in the maintenance of EHRs by continuing to receive their health information through existing medical information systems and then upload their health data to blockchain-based EHRs through them. This method, with the “minimum unit” as the main body, has high operability but relies on the patient’s additional operational steps as an intermediate link. This solution poses a high risk to data integrity, and if the patient does not complete the manual upload step, it will result in incomplete records. The algorithm for generating symmetric keys K from the user-specified security parameter p is KeyGen(p)→K, where KeyGen represents the cryptographic key generator. Both EHR file encryption and index encryption are completed with two different symmetric keys, as follows:

(1) $${KeyGen}_{ENC}(p_{1},p_{2})\to (K_{1},K_{2})$$

where ENC represents the searchable symmetric encryption and $p_{1},{\;p}_{2}$ and $K_{1},K_{2}$ are symbolized randomly generated passphrases and searchable symmetric encryption keys, respectively.

### 5.2. Maintaining Health Data Security

Personal health information belongs to the private sector and is more valuable than other personal information. Data leakage in the medical industry is more dangerous than in any other industry. In recent years, the continuous occurrence of customer privacy breaches has continuously consumed users’ trust in third-party service platforms [61,62], and people urgently hope to explore a new way to replace centralization. The server for the blockchain provides a more secure solution for data storage. The information in the blockchain database is encrypted using asymmetric encryption technology, and each user has a unique key pair consisting of a public key and a private key [63]. On the blockchain platform, archive data are broadcast and stored on the blockchain network in the form of an index, archive type, and digital fingerprint (hash function). For two-way verification to work, both the EHR owner and the cloud service provider must create a pair of elliptic curve digital signature algorithm public and private keys and start a hash function, as follows:

(2) $${KeyGen}_{ECDSA}(User)\to ({PK}_{U},{SK}_{U})$$

(3) $${KeyGen}_{ECDSA}(CSP)\to ({PK}_{S},{SK}_{S})$$

where CSP represents the cloud service provider; ${PK}_{U},{SK}_{U}$ signify the user’s public and secret key; ECDSA characterizes the elliptic curve digital signature algorithm; and ${PK}_{S},{SK}_{S}$ mean the server’s *ECDSA* public and secret key.

After setting up all of the necessary safeguards, the EHR’s rightful owner will move and launch the smart contracts on the blockchain. In exchange, the owner will be given the smart contract metadata necessary to interact with the contracts on the blockchain.

Following the encryption of the EHR, the proprietor proceeds to upload the resulting ciphertext, denoted as $C_{EHR}$, onto the inter planetary file system (IPFS) for the purpose of outsourcing storage. The construction of bitmap index *M* is finalized by the proprietor of the EHR through the insertion of the file hash into the initial row, serving as the unique identifier for the document. The computation of the encryption for both the *EHR* and the bitmap matrix is as follows:

(4) $${ENC}_{K_{1}}(iv,EHR)\to C_{EHR}$$

(5) $${ENC}_{K_{2}}(iv,M)\to C_{M}$$

**Figure 4:**
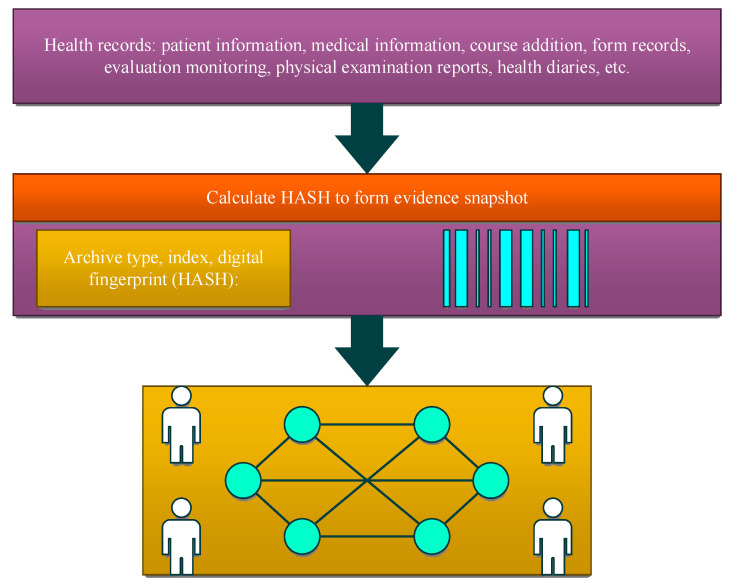
Schematic map of data storage process.

IPFS receives *EHR* ciphertext $C_{EHR}$. The blockchain distributes the bitmap index ciphertext $C_{M}$. The EHRs platform also strengthens security controls over data transmission, storage, authority control, etc. All data access and transmission use secure channels. The database on the blockchain platform does not store plaintext data. The encryption key is on the hardware encryption machine. The database administrator (DBA) and application operation and maintenance, as well as the encryption machine operation and maintenance permissions, are separated, and no one can obtain plaintext data, as shown in Figure 4.

### 5.3. Implement Differentiated Authorization Management

**Figure 5:**
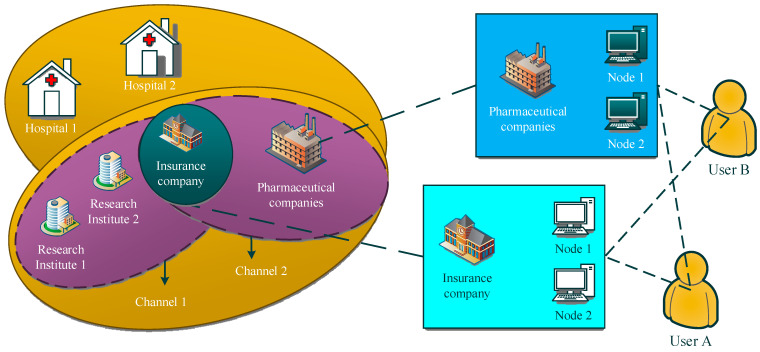
Schematic diagram of data differentiation management.

Medical health data are tremendously complex from both a personal and legal standpoint, so patients should be the ones to hold it. In addition, patient-centered EHRs are also beneficial for maintaining the single authenticity of health data. Hyperledger fabric is a licensed blockchain platform with differentiated features that provide extremely high scalability for licensed blockchains [64]. Participants on the fabric network can establish a “channel” between subsets of participants that is granted visibility to a specific transaction set. Multiple different organizations on the fabric blockchain can form alliances. Several different organizations under the alliance have established separate channels, each with an independent distributed ledger. This channel connecting participants and their subsets is granted specific transaction set visibility. The channel mechanism can ensure the formation of a proprietary private network between member organizations, where only organizational members within the channel can share accounting books. Private data are effectively isolated from external, unrelated organizations or individuals, as shown in Figure 5.

The blockchain application system based on Hyperledger fabric achieves differentiated management of personal medical data by assigning a set of access permissions, specifying who can query and write data to their blockchain, as well as the duration. Patients can provide different levels of access permission to different institutions or personnel. This enables patients to interact with community health service centers, hospitals, research institutes (laboratories), pharmaceutical companies, insurance companies, and other institutions to varying degrees within appropriate ranges through differentiated authorization. In addition, the blockchain application system based on Hyperledger fabric also connects research institutions with patients willing to share health data for research in the health data market. After the research institution clearly informs patients of the type and purpose of data they need, patients can provide partial health data authorization, which provides convenience for the research institution. Patients will also receive corresponding economic compensation, thereby benefiting from the potential value of health data.

## 6. Results Analysis

In order to accurately reflect the medical process, improve medical quality and safety, and safeguard the rights and interests of both doctors and patients, medical institutions have formulated strict medical record management systems. Blockchain and the IoT are two technologies that have the potential to revolutionize the healthcare industry. The integration of these two technologies can create an e-healthcare system that is more secure, efficient, and transparent. In the era of “e-healthcare”, the implementation of new concepts such as telemedicine and internet hospitals urgently need an internet-based medical record management system.

**Figure 6:**
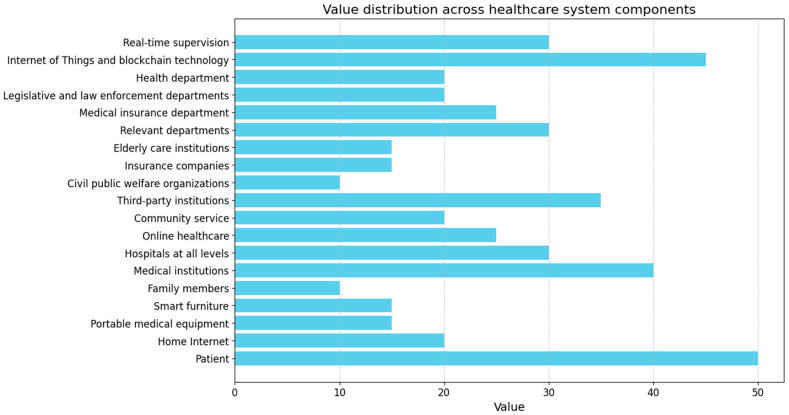
Considering the division of value among e-healthcare system components, where the Internet of Things and blockchain technology value is 45. This component holds the second highest level of significance, highlighting the increasing importance of technology in contemporary healthcare.

Blockchain has created a new trust mechanism with distributed storage, smart contracts, timestamps, and other technological features protecting the integrity and reliability of medical behavior and data. The present section evaluates the security stage and performance of the proposed “e-healthcare” management scheme, which is a blockchain- and IoT-based healthcare management system that incorporates two-side verifiability. The analysis of security pertains to the characteristics that may be fulfilled in the suggested framework for attaining the objectives of security. The evaluation of performance aims to analyze the efficiency of the system by assessing the results related to performance in the implementation of e-healthcare. The feature comparison section entails a thorough examination of the features of e-healthcare in contrast to the various solutions put forth in the extant literature. Figure 6 indicates the distribution of values among different components in a healthcare system. Every element is allocated a numerical value that signifies its significance, impact, or contribution to the entire system.

The “Patient” node possesses the utmost value in the network, as anticipated, since the patient serves as the central focal point of the healthcare system. Every other element is ultimately focused on enhancing patient results and delivering efficient healthcare. “Medical institutions”, such as hospitals, online healthcare platforms, and community programs, have a crucial function in providing healthcare services. Their significant worth emphasizes their crucial role in the system, delivering hands-on care and medical interventions. The significance of “online healthcare” services has grown significantly, especially in light of the COVID-19 epidemic. The value represents their contribution to expanding healthcare access to a wider population through remote and easily accessible solutions. The allocation of value among the many components of the healthcare system highlights the key role of the patient and the growing significance of technology in healthcare. Medical institutions, related departments, and third-party institutions are crucial, but emerging technologies like the IoT and blockchain are becoming essential. This study emphasizes the necessity of a well-rounded and integrated system in which every component has a role in enhancing patient care and achieving desired outcomes.

The efficiency of the proposed searchable encryption scheme was evaluated through testing of the implemented system. The use of blockchain and IoT technologies in healthcare can help to secure patient data by providing a tamper-proof record of all transactions. This can help to prevent data breaches and ensure that patient data are kept private and confidential. Figure 6 illustrates the outcomes acquired from the encryption process of various dimensions of EHR. Typically, the EHR of a patient is expected to expand as additional diagnoses or treatments are administered over a period of time. Medical screening produces a substantial volume of data, including the results of imaging techniques such as X-ray, CT scan, and ultrasound imaging. These data are utilized for illustrative purposes. Accordingly, this particular metric has the ability to accurately represent and emulate the practical application of EHR documentation.

**Figure 7:**
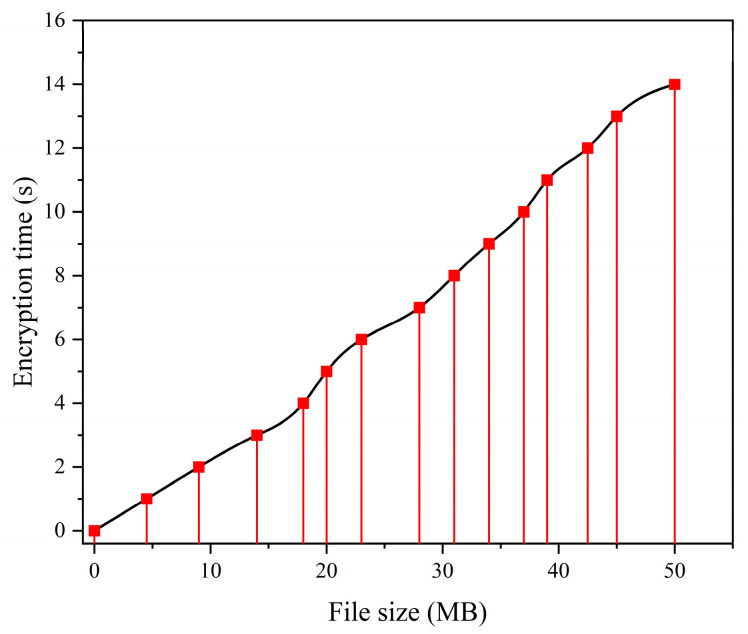
The encryption time of EHRs as a function of various file sizes.

Figure 7 shows a positive correlation between the size of EHRs in megabytes (MB) and the time required for encryption. As a design, it is noteworthy that a file with a size of 10 MB necessitates an encryption duration of under 3 s. In contrast, a file with a size of 50 megabytes necessitates approximately 14 s of encryption time. This means the curvature is very close to being straight. The e-healthcare management encoding system utilizes the efficient advanced encryption standard cipher block chaining encryption mode, which allows for database encryption of moderate to large sizes without requiring an impractically long time to decode. Consequently, the curve exhibits a nearly linear behavior. The blockchain- and IoT-based e-healthcare encryption scheme utilizes an AES-CBC (advanced encryption standard–cipher block chaining) encryption mode that demonstrates high efficiency in supporting encryption for databases of medium to large sizes while avoiding exponential increases in encryption time.

Table 3 presents a comparison of the encryption time among the schemes introduced by Wang S. et al. [63], Wang H. et al. [65], and the proposed scheme. The columns denote the magnitude of the file in kilobytes (KB), whereas the rows represent the duration of encryption in seconds (s). As the comparative methodologies have not been re-implemented, the outcomes are cited and derived from the primary research article. The file subject utilized for comparison purposes is restricted to a maximum size of 1200 kilobytes, or approximately 1.2 MB, in order to maintain consistency with the analysis of similar file sizes employed in the aforementioned two schemes. The comparative analysis suggests that the encryption algorithm implemented in blockchain- and IoT-based e-healthcare exhibits superior efficiency in comparison to the other methodologies. It can be observed that, in comparison to other schemes, the encryption time exhibits a relatively lower increase as the size of the file being processed increases. Therefore, the solution we propose offers a more expedient encryption performance that is both practical and future-proof for implementation in a large-scale EHR storage and sharing system.

From the table, the encryption efficiency of the proposed system is compared against other systems (such as the SWIPT CR system and the cloud-based EHR system). The table provides comparative data on encryption times for different file sizes, indicating that the proposed system performs significantly faster in all cases. There is also a graph depicting encryption time as a function of file size, showing that the system’s performance scales linearly with increasing file sizes. The findings indicate that our proposed e-healthcare exhibits superior performance in comparison to other ciphers. In the cross-hospital and cross-regional flow of medical data, the medical data involved, such as EHRs, are not only electronic records of the patient’s course but also credible digital evidence with legal effect. This outcome may be attributed to various factors such as the cipher’s implementation environment and modes of operation. The comparing schemes’ system implementations were executed in a computing environment featuring an Intel Core (TM) i5-10210U processor and 24 GB of random-access memory (RAM), for reference purposes. The two schemes were implemented using the Java programming language. In contrast, the implementation of our system was carried out utilizing JavaScript programming language. EHRs and other medical records are stored on the blockchain, and any modification of medical records will be clearly marked, dated, and signed. Consequently, the outcome is contingent upon the CPU specifications, the choice of programming language, and the encryption algorithm utilized. Furthermore, the efficacy of our proposed scheme and the scheme presented by Wang S. exhibits a notable increase in comparison to the scheme proposed by Wang and Song, particularly in scenarios where the file size is considerable. The reason for this is that the methodology employed by Wang S. involves the utilization of an attribute-based encryption (ABE) algorithm that entails higher computational costs and necessitates a lengthier encryption duration. This comparative analysis demonstrates the overall superiority in performance of symmetric ciphers over asymmetric ciphers, exemplified by the ABE algorithm. Table 4 compares the proposed system with the SWIPT CR system and cloud-based EHR system across several performance aspects such as security, efficiency, scalability, and encryption speed.

This table outlines how the proposed system compares favorably in terms of encryption speed, scalability, and resource usage while offering high security and minimal latency. The proposed system outperforms the other systems, especially in high data load and resource-efficient encryption. The comparison table highlights the performance of the proposed system against the SWIPT CR system and cloud-based EHR system across various key metrics. The proposed system significantly outperforms both existing systems in terms of encryption speed, with an encryption time of just 0.1 s for a 1 MB file, compared to 1.8 s for the SWIPT CR system and 7 s for the cloud-based EHR system. Similarly, for larger file sizes (10 MB), the proposed system maintains an encryption time of 0.2 s, while the other systems experience considerable delays.

In terms of scalability, the proposed system demonstrates high efficiency, handling datasets up to 1 GB and beyond, while the SWIPT CR and cloud-based EHR systems are limited to smaller datasets of 100 MB and 200 MB, respectively. The proposed system also supports advanced encryption methods like AES-256, ensuring stronger security without a significant impact on performance. Furthermore, the proposed system exhibits minimal latency under high data loads, making it ideal for real-time applications, whereas the other systems suffer from moderate to significant latency increases.

Another notable advantage is in resource efficiency. The proposed system has low CPU and memory consumption, in contrast to the high resource usage observed in the SWIPT CR system and cloud-based EHR system. In terms of throughput, the proposed system can process 200 files per second, a drastic improvement over the 50 and 20 files per second capabilities of the SWIPT CR and cloud-based EHR systems, respectively. Overall, the proposed system shows remarkable adaptability to varying security requirements and scales efficiently, making it a superior solution for both security and performance.

The majority of the examined methodologies fail to account for the provision of assistance towards the dynamic updating of EHRs. The proposed framework by Chelladurai et al. [41] incorporates the consideration and mention of the EHR update functionality. Nevertheless, this particular scheme is deficient in several crucial security aspects. The blockchain- and IoT-based e-healthcare scheme under consideration facilitates dynamic updates through the implementation of a bitmap index. The dynamic nature of the bitmap index matrix allows for modifications or additions to the EHR without necessitating the rebuilding of the index. The bitmap index can be readily updated through the creation of additional rows and columns to accommodate newly uploaded data. Blockchain-based e-healthcare systems can track the entire lifecycle of pharmaceutical products, ensuring the authenticity and integrity of drugs. By integrating IoT sensors and devices into the supply chain, healthcare providers can monitor the storage conditions, transportation, and delivery of medications, reducing the risk of counterfeit drugs and enhancing patient safety.

## 7. Conclusions

Considering the long-standing inertia and investment situation in the healthcare industry, it is unlikely that the industry will easily accept a shift towards an unproven new technology at this stage. Therefore, this study proposes a transitional solution compatible with infrastructure, integrating the current “e-healthcare” construction with blockchain and IoT technology. In the medical industry, blockchain technology may be the key to improving data availability and interoperability among medical service providers, patients, insurance companies, and medical researchers. The e-healthcare management system based on blockchain and the IoT has effectively solved the existing problems:

(1) By combining portable medical equipment and other devices with the IoT and blockchain, the authenticity and reliability of patients’ vital signs, treatment, medication status, and other data are ensured, reducing medical risks and alleviating the limitations of the medical environment.

(2) E-healthcare management systems based on big data solve the problem of medical resource shortages by enabling patient information to be quickly synchronized at all levels of hospitals, allowing patients to seek treatment efficiently while undergoing cross-hospital and cross-department treatments.

(3) Multiple institutions on the blockchain can jointly participate in supervision to achieve efficient claim settlement. In medical insurance, the diagnosis and treatment data and medical treatment status of patients become the keys to claim settlement. The e-healthcare management system collaborates with patients, hospitals, third-party companies, relevant departments, and other institutions to jointly review and verify the medical and medical treatment data of patients and achieve rapid claim settlement according to the agreement.

(4) E-healthcare management systems can transform medical credit data into digital assets, improve the issue of medical data fraud, and ensure the authenticity of medical data while simultaneously reducing construction and operational costs, solving the problem of difficult certification in medical accidents, and improving the operational efficiency of the medical system.

Nevertheless, the e-healthcare management system can provide patients with one-stop services such as emergency calls, public welfare organizations, medical insurance institutions, medical services, etc. Using the IoT and blockchain technology, a health and medical big data traceability system has been established in collaboration with medical and medical insurance institutions. This has resulted in the achievement of reliable traceability of medical equipment tracking, drug tracking, patient health and medical big data, and medical insurance data [66], which has enabled the improved development of home medical systems. However, there is no consensus on the potential value of this new technology. Some scholars believe that blockchain technology may bring greater disruptive changes than the internet, while others question its importance. The feasibility and availability of the application of blockchain and IoT technology to “e-healthcare” depends on the common development of technology maturity, stakeholders, national policies and regulations, and other factors.

## Figures and Tables

**Table 1:** Blockchain and IoT healthcare assessments.

No.	References	Advantages	Disadvantages
1	2019 [30]	The authors outlined potential uses for blockchain technology in medical settings.	Issues and potential remedies were not prioritized.
2	2019 [31]	The article focused on blockchain for the Internet of Things	-
3	2019 [32]	They conducted a review of the cyber security measures implemented in blockchain technology.	However, it is important to note that the scope of the discussion does not specifically pertain to the healthcare industry.
4	2020 [33]	To present an identity system for patients based on blockchain technology in the hospital setting.	-
5	2020 [34]	To conduct a review of healthcare systems that are based on the IoT, discussing their potential applications, difficulties, and challenges.	On the other hand, numerous papers have recently offered possible ways for implementing blockchain technology in healthcare, which does not address.
6	2020 [35]	This report provided a comprehensive literature assessment of healthcare blockchain systems from 2016 to January 2020.	Hence, there is a need for a novel review article to deliberate on the present challenges and remedies.
7	2021 [36]	A recent literature review that focused on the use and difficulties of blockchain in the IoT.	The urgent problems in healthcare were not addressed in depth by the writers.
8	2021 [37]	The primary focus of the literature was to underscore the attributes and traits of blockchain technology in the context of managing healthcare data.	Nonetheless, the authors failed to concentrate on the mechanics of blockchain technology and its potential to mitigate the drawbacks of the traditional healthcare system.
9	2022 [38]	The present study aims to examine the applications and challenges of blockchain technology in the healthcare system, specifically in the context of IoT-enabled systems.	This study does not discover and assess their functions to increase blockchain scalability in many sectors.
10	2023 [39]	It points the way for additional study of the cryptographic properties of blockchain applications within the context of the Internet of Medical Things.	It is imperative to establish a suitable framework and implement rigorous oversight mechanisms.

**Table 2:** List of resources for e-health evaluation.

No.	References	Existing Resource	Hub	Apply for
1	Kuan et al. [48] Martin et al. [49]	National Health Service	Attempt at validation supported by the government and carried out by a private enterprise, Our Portable Medical Care.	Clinical effectiveness, regulatory compliance, clinical health, confidentiality and safety of data, protection, usability and accessibility, user testing, compatibility, technical stability, and managing change are all important considerations.
2	Chan et al. [50] Jones et al. [51]	Wellocracy	Apps and devices for fitness, good eating, sleeping well, managing stress, and maintaining a healthy cardiovascular system.	Analytical reviews of the mobile applications available in each category, including consumer feedback in the form of relative comparisons. The most important aspects are the “Fun Features”, “Which it Had”, compatibility (device, iOS vs. Android), and customer reviews.
3	Mathews et al. [52]	Personal Connected Health Alliance	Data standards for connected devices and mobile platforms; further development of FHIR, access to the cloud, and cyber security.	Interfaces for medical devices, healthcare services, and computerized medical records
4	Karsalia et al. [53]	Xcertia	Generalized selection standards for mobile apps. Separate teams were formed to address concerns about privacy, usability, evidence-based information, and promotion.	Accessibility, usability, data protection, and confidentiality
5	Dang et al. [54] Hong et al. [55]	Digital therapeutics	Optimized medicines and digital health solutions for the prevention, diagnosis, and treatment of medical conditions. Mostly within the confines of established rules.	Not a grading system, but a set of principles to be adopted, such as those related to drug improvement, clinical confirmation, security and safety, and encouraging proper regulatory scrutiny of product claims and dangers.

**Table 3:** Encryption efficiency comparison.

Model	File Size (kB)						
	0	200	400	600	800	1000	1200
SWIPT CR system [63]	0 s	1.8 s	3 s	4 s	6 s	8 s	10 s
Cloud-based EHR system [65]	0 s	7 s	15 s	20 s	26 s	32 s	35 s
Proposed work	0 s	0.1 s	0.2 s	0.2 s	0.2 s	0.3 s	0.3 s

**Table 4:** Comparing the proposed system with the SWIPT CR system and cloud-based EHR system across different performance factors.

Performance Metric	SWIPT CR System	Cloud-Based EHR System	Proposed System
Encryption Speed (for 1 MB file)	1.8 s	7 s	0.1 s
Encryption Speed (for 5 MB file)	4 s	20 s	0.2 s
Encryption Speed (for 10 MB file)	6 s	26 s	0.2 s
Scalability	Moderate scalability (up to medium data loads)	Low scalability (slows down significantly with larger datasets)	High scalability (handles large datasets efficiently)
Security Options	AES-128	AES-128, AES-256	AES-256, advanced encryption methods
Energy Efficiency	Moderate	Low	High
Latency Under Load	Moderate increase in latency	Significant increase in latency	Minimal increase in latency
Resource Usage (CPU/Memory)	High CPU and memory usage	High CPU, moderate memory	Low CPU and memory consumption
Support for Large Datasets	Up to 100 MB	Up to 200 MB	Up to 1 GB and beyond
Throughput (files per second)	50 files/s	20 files/s	200 files/s
Adaptability to Security Requirements	Limited to pre-defined security	Adaptable, but less efficient at higher security levels	Highly adaptable with minimal performance loss

## Data Availability

All the data are in the manuscript.
